# Predictors of Respiratory Syncytial Virus, Influenza Virus, and Human Metapneumovirus Carriage in Children Under 5 Years With WHO‐Defined Fast‐Breathing Pneumonia in Pakistan

**DOI:** 10.1111/irv.13285

**Published:** 2024-04-14

**Authors:** Muhammad Imran Nisar, Salima Kerai, Shahira Shahid, Muhammad Farrukh Qazi, Sarah Rehman, Fatima Aziz, Fyezah Jehan

**Affiliations:** ^1^ Department of Pediatrics and Child Health Aga Khan University Karachi Pakistan; ^2^ School of Population and Public Health University of British Columbia Vancouver British Columbia Canada

**Keywords:** human metapneumovirus, influenza virus, nasopharyngeal carriage, Pakistan, respiratory syncytial virus

## Abstract

**Background:**

Pneumonia is a leading cause of morbidity and mortality in children < 5 years. We describe nasopharyngeal carriage of respiratory syncytial virus (RSV), human metapneumovirus (hMPV), and influenza virus among children with fast‐breathing pneumonia in Karachi, Pakistan.

**Methods:**

We performed a cross‐sectional analysis of nasopharyngeal swabs from children aged 2–59 months with fast‐breathing pneumonia, enrolled in the randomized trial of amoxicillin versus placebo for fast‐breathing pneumonia (RETAPP) (NCT02372461) from 2014 to 2016. Swabs were collected using WHO standardized methods, processed at the Aga Khan University, Pakistan. Viral detection was performed using LUMINEX xTAG respiratory viral panel assay and logistic regression identified clinical and sociodemographic predictors.

**Findings:**

Of the 1000 children tested, 92.2% (*n* = 922) were positive for viral carriage. RSV, hMPV, and influenza virus were detected in 59 (6.4%), 56 (6.1%), and 58 (6.3%) children and co‐infections in three samples (two RSV‐hMPV and one influenza‐hMPV). RSV carriage was common in infants (56%), we observed a higher occurrence of fever in children with hMPV and influenza virus (80% and 88%, respectively) and fast breathing in RSV (80%) carriage. RSV carriage was positively associated with a history of fast/difficulty breathing (aOR: 1.96, 95% CI 1.02–3.76) and low oxygen saturation (aOR: 2.52, 95% CI 1.32–4.82), hMPV carriage was positively associated with a complete vaccination status (aOR: 2.22, 95% CI 1.23–4.00) and body temperature ≥ 37.5°C (aOR: 2.34, 95% CI 1.35–4.04) whereas influenza viral carriage was associated with body temperature ≥ 37.5°C (aOR: 4.48, 95% CI 2.53–7.93).

**Conclusion:**

We observed a high nasopharyngeal viral carriage among children with WHO‐defined fast‐breathing pneumonia in Pakistan. Fever, difficulty in breathing, hypoxia and vaccination status are important clinical predictors for viral nonsevere community‐acquired pneumonia.

## 1 Background

Community‐acquired pneumonia (CAP) is one of the leading causes of morbidity and mortality in children under 5 years worldwide [1]. Low‐ and middle‐income countries (LMICs) share two‐thirds of the disease burden, accounting for 0.29 episodes of pneumonia per child annually [2, 3]. The introduction of vaccines against pneumococcus and *Haemophilus influenzae* Type b (HiB) has significantly reduced the incidence and case‐fatality rates of pneumonia by more than a third during the past decade [4]. However, Pakistan reported a 50% increase in the incidence of CAP from 195 to 289 new episodes per 1000 children from 2000 to 2015 [4]. The Pneumonia Etiology Research for Child Health (PERCH) study reported that viral etiologies accounted for 61.4% of the severe pneumonia cases in children less than 5 years in LMICs [5]. Respiratory syncytial virus (RSV), parainfluenza virus, and human metapneumovirus (hMPV) were among the top 10 etiologies of severe pneumonia in this age group [5]. The emergence of modern nucleic acid–based molecular diagnostic methods currently allows for the detection of viral respiratory pathogens with greater specificity and sensitivity than previously possible [6].

The Infectious Diseases Society of America guidelines recommend viral testing of nasopharyngeal secretions and/or nasal swabs by PCR or immunofluorescence for the management of childhood pneumonia [7]. Nasopharyngeal colonization is considered a prerequisite for *Streptococcus pneumoniae* infections, which account for the highest burden in Pakistan [8]. Colonization can progress to either invasive or noninvasive disease such as pneumonia, sepsis, meningitis, and otitis media, and most of the episodes are caused by a limited number of serotypes [9]. In Pakistan, 80% of the children less than 5 years carried pneumococcus, and only 12.1% of these were responsible for the disease‐causing serotypes in the post‐PCV10 period [10]. In addition, the PERCH study findings showed that viruses (RSV, parainfluenza 1, and hMPV), when found in nasopharyngeal secretions, were likely to be a cause of severe pneumonia [5].

However, low‐resource settings such as Pakistan do not have viral molecular assays available. In such settings, the Integrated Management of Childhood Illness (IMCI) provides a syndromic management approach based on which the service provider follows various clinical algorithms to classify and treat childhood illnesses according to their severity [11]. The IMCI‐based pneumonia classification involves the evaluation of respiratory rate, a critical step that influences the management and referral decisions. The classification is made into three categories: cough and cold, pneumonia (with chest indrawing or fast breathing), and severe pneumonia (with danger signs or stridor). The second category is managed with oral antibiotics in primary healthcare and home settings, while the third requires referral to a secondary center for parenteral antibiotics and ongoing monitoring [11]. Despite its high sensitivity, only a quarter of the children are correctly identified by community workers using IMCI, and its inability to distinguish between viral and bacterial cases further complicates diagnosis [5]. Thus, the IMCI can be informed by clinical characteristics specific to common viral pneumonia etiologies such as the RSV, influenza virus, and hMPV responsible for a majority of the viral pneumonia in Pakistan [12].

In this study, we aim to assess the carriage prevalence of RSV, influenza virus, and hMPV among a cohort of children ages less than 5 years with fast‐breathing pneumonia in a peri‐urban community setting in Karachi, Pakistan, and explore their association with clinical and sociodemographic predictors.

## 2 Methods

### 2.1 Study Design and Participants

We conducted a cross‐sectional analysis of a subsample of children enrolled in the RETAPP trial (Randomized Trial of Amoxicillin Versus Placebo for [Fast‐Breathing] Pneumonia) (NCT02372461), a double‐blind, randomized, controlled noninferiority trial comparing placebo with amoxicillin for the management of World Health Organization (WHO)–defined fast‐breathing pneumonia [13]. The study was carried out from November 9, 2014, to November 30, 2017, in a peri‐urban slum area, Bin Qasim town, in Karachi, Pakistan, where the Aga Khan University has provided demographic surveillance, along with primary healthcare centers for managing common childhood illnesses. The study area constituted 28,000 children under the age of 5 years. The incidence of pneumonia was reported to be 25 cases per 100,000 child‐years, with a case fatality rate of 1.5% [14]. We enrolled children aged 2–59 months seeking care at primary health centers with a (observed or reported) history of cough or difficult breathing within the past 14 days and a respiratory rate ≥ 50 breaths per minute (for ages 2 to < 12 months) or ≥ 40 breaths per minute (for ages ≥ 12 months) on two consecutive readings. In addition, children who presented with wheeze along with cough or difficulty in breathing were given a trial of nebulization with a bronchodilator up to three times, 15–20 min apart, and the respiratory rate was assessed after each nebulization therapy. If the respiratory rate remained persistently above the cutoff, irrespective of wheeze, the child was considered for inclusion after written informed consent was obtained from the legal guardians.

We excluded children with recent antibiotic use within the previous 48 h, lower chest wall indrawing (a criterion for severe pneumonia in the guideline before 2014), any general danger sign defined by the World Health Organization, bulging fontanel, pedal edema, known cases of asthma, tuberculosis, or other severe illnesses, a history of hospitalization in the last 2 weeks, congenital heart disease, surgical conditions requiring hospitalization, being out of the catchment area, and concurrent enrollment in another trial or previous participation within the last 6 months. Further details regarding study methods have been published elsewhere [15].

Each child's baseline information regarding sociodemographics, air quality indicators, birth, breastfeeding, and immunization history were collected. Clinical history, physical examination, and anthropometry were recorded. Any deterioration detected either by a CHW during home visit (including borderline oxygen saturation defined as ≤ 92%) or by family at any time was promptly reported on a hotline number to a 24/7 on‐call physician with facilitated referral to a tertiary care hospital if required.

A trained female health worker and a physician independently assessed respiratory rates, designating a child with tachypnea if there was agreement. Wheeze was assessed through auscultation by a study physician, all children with wheeze received up to three doses of inhaled bronchodilator according to WHO‐IMCI guidelines for categorization of fast breathing with wheeze [11]. After each bronchodilator use, respiratory rate was recounted, and child was recategorized for fast breathing. Only children having persistent fast breathing after a maximum of three inhalations were considered for enrollment irrespective of wheeze. This was done to remain in alignment with IMCI guidelines [11]. Children were then randomized to receive oral amoxicillin or a pharmacologically inert placebo, followed by the collection nasopharyngeal swabs for the detection of viral pathogens.

### 2.2 Nasopharyngeal Swab Collection and Analysis

Nasopharyngeal swabs were collected by trained community health workers after obtaining written informed consent from legal guardians. We followed the WHO's consensus methods for swab collection [16]. The samples were collected in a commercially available Universal Transport Medium (UTM, Diagnostic Hybrids Inc.) and transported at 2°C–8°C to the Infectious Diseases Research Laboratory at Aga Khan University in Karachi where they were stored in −80°C freezers for further molecular assay testing.

Frozen samples were thawed and spiked with MS2 bacteriophage (external control) before total nucleic extraction to check the efficiency of nucleic acid amplification. We performed total nucleic acid extraction in 400 μL of the specimen using MagNA Pure Compact Nucleic Acid Isolation Kit I (Roche Life Science, Indianapolis, IN, USA). Extracted nucleic acids were eluted in 100 μL of elution buffer. The xTAG respiratory viral panel (RVP) fast assay kit (Luminex 200 Molecular Diagnostic Inc., Toronto, Canada) detected a wide range of viruses and their subtypes, including influenza A and subtypes (nonspecific influenza A, H1, H3, and 2009 H1N1), influenza B, RSV, parainfluenza (Types I–IV), hMPV, adenovirus, enterovirus/rhinovirus, coronavirus (NL63, HKU1, 229E, and OC43), and human bocavirus following the manufacturer's instructions. We analyzed and reported data as median fluorescent intensity using TDAS RVP FAST software, version 2.2.

### 2.3 Statistical Analysis

We reported the clinical, and sociodemographic characteristics of children with different viral carriages as frequencies and percentages. Viral carriage was defined as the percentage of samples that tested positive for a virus out of the total number of positive samples in the study. Fast‐breathing pneumonia was defined as a respiratory rate of at least 50 breaths in an infant aged 2–11 months or at least 40 breaths or more in child 12–59 months with no chest indrawing. Influenza virus prevalence indicated both influenza A and subtypes (nonspecific influenza A, H1, H3, and 2009 H1N1) and influenza B. A body temperature equal to or above 37.5°C was defined as fever. Anthropometric measurements were converted to *Z*‐scores for weight‐for‐age (WAZ), height‐for‐age (HAZ), and weight‐for‐height (WHZ) based on WHO cutoffs [17]. A cutoff of −2 *Z*‐scores for these indices was used for classifying children for wasting (WHZ < −2SD), underweight (WAZ < −2SD), and stunting (HAZ < −2SD). Anemia was assessed by the presence of palmar pallor, categorized as some palmar pallor or severe palmar pallor. Air quality indicators included regularly open windows for ventilation, number of people who sleep in the same room categorized as ≤ 3 or >3, presence or absence of pets in the household, and a smoker in household. A fully vaccinated child had received all the age‐appropriate vaccines as defined by the Federal Directorate on Immunization (FDI), Pakistan. The primary scheme of vaccination in Pakistan includes the Bacille Calmette‐Guérin (BCG) vaccine at birth, four doses of the oral polio vaccine (OPV0) administered at birth, 6, 10, and 14 weeks. Three doses of the pentavalent vaccine, which includes the combined diphtheria, tetanus, and pertussis (DPT) antigens, hepatitis B vaccine (HBV), and HiB vaccines administered at 6, 10, and 14 weeks. Additionally, three doses of the 10‐valent pneumococcal conjugate vaccine (PCV10) are given at 6, 10, and 14 weeks, along with two doses of the rotavirus vaccine at 6 and 10 weeks of age. This is followed by two doses of measles at 9 and 15 months.

Logistic regression analysis was used to identify clinical or sociodemographic predictors of three common/selected viruses: RSV, hMPV, and influenza virus among children with fast‐breathing pneumonia. The final model was adjusted for sex, age categories, maternal education, exclusive breastfeeding for 6 months, vaccination status, history of symptoms, clinical signs, anthropometric measurements, number of household members sharing a room, pets in household, current smoker in household, type of fuel and stove used for cooking, and presence of child near cooking area.

For the purpose of model building, all variables with a *p*‐value less than 0.25 in the bivariate analysis were used to build a multivariable model. A backward selection procedure was used to derive a parsimonious model for retaining only variables significant at *p*‐value ≤ 0.05. All analyses were performed using STATA version 12.0.

### 2.4 Ethics

Ethics approval was given by Ethics Review Committee of Aga Khan University Pakistan (ERC reference number 2786‐Ped‐ERC‐13) with additional endorsement from the Faculty of Medicine Ethics Committee at Southampton University, UK. All parents or legal guardians provided written, informed consent obtained by a nonstudy physician.

## 3 Results

**FIGURE 1:**
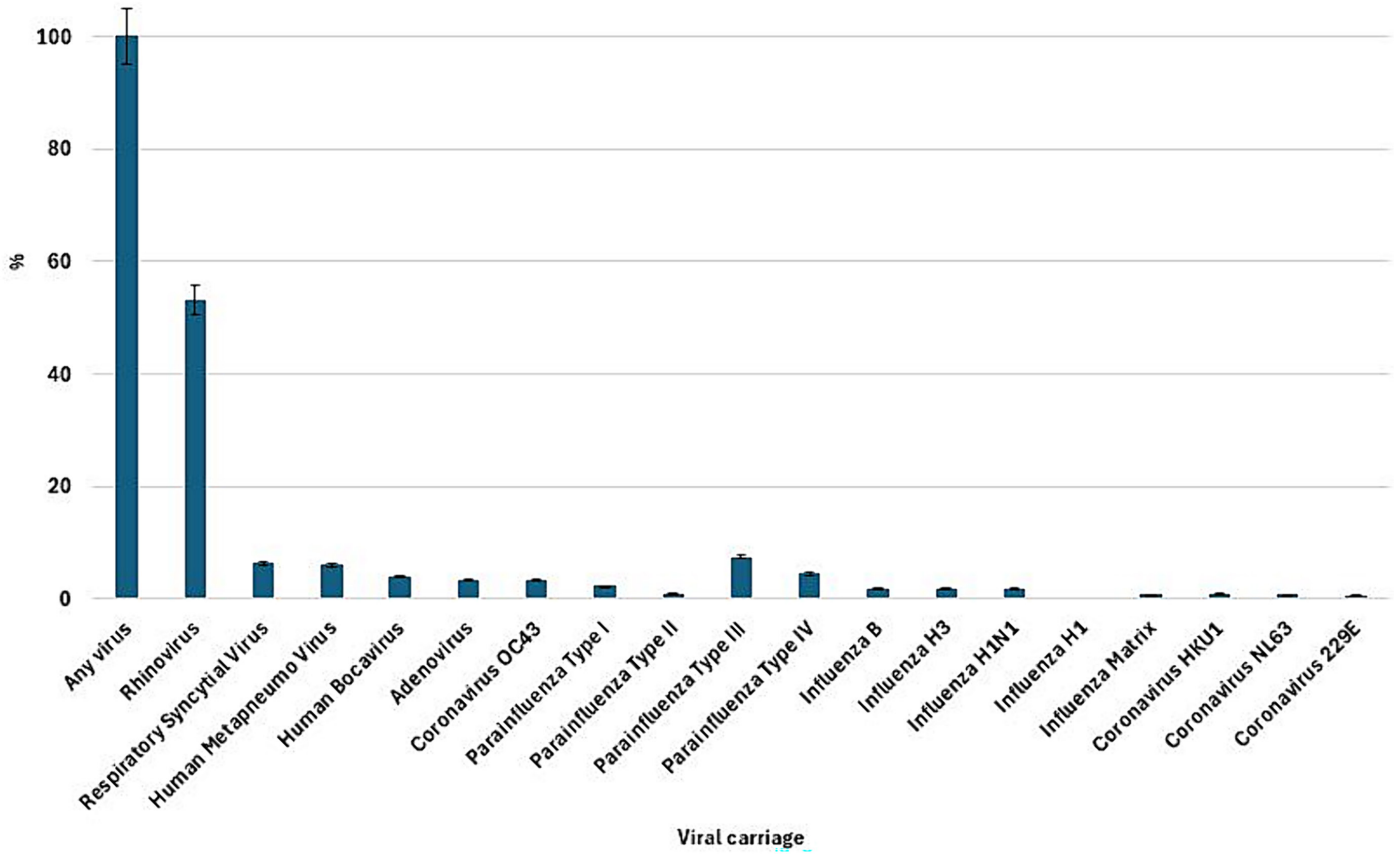
Distribution of viral infection among children with fast‐breathing pneumonia (*n* = 1000).

We enrolled 1000 children in the study, and 92.2% (*n* = 922) of their samples were positive for viral carriage. The most prevalent virus was rhinovirus (53.1%, *n* = 490/922). RSV was detected in 59 (6.4%) children, hMPV in 56 (6.1%), and influenza virus in 58 (6.3%%) children, of which the most prevalent was influenza B virus (*n* = 18). Coinfection was detected in three samples (two RSV‐hMPV and one influenza virus and hMPV) (Figure 1). Table 1 describes the demographic and clinical characteristics of all children and those with RSV, hMPV, and influenza virus carriage. Female children predominantly carried hMPV (62%), followed by RSV (59%) and influenza (55%) virus in their nasopharynx. RSV was more frequently detected in infants (2–11 months) (56%), while hMPV and influenza virus in children (12–59 months) (63% and 67%, respectively). More than half of the children with RSV (56%), hMPV (63%), and influenza (52%) virus carriage were exclusively breastfed. Almost half of the children with RSV, hMPV, and influenza viral carriage had received all age‐appropriate vaccines (42%, 50%, and 41%). A majority (68.2%) of the samples was collected in 2015 (Table 2).

**TABLE 1:** Demographic and clinical characteristics of children with viral carriage.

Characteristics	Total	Respiratory syncytial virus	Human metapneumovirus	Influenza virus
	*n* = 1000	*n* = 59	*n* = 56	*n* = 58
Sex				
Female	489 (49%)	35 (59%)	35 (63%)	32 (55%)
Male	511 (51%)	24 (41%)	21 (38%)	26 (45%)
Age categories (in months)				
2–11	444 (44%)	33 (56%)	21 (38%)	19 (33%)
12–59	556 (56%)	26 (44%)	35 (63%)	39 (67%)
Maternal years of schooling				
No education	562 (56%)	36 (61%)	24 (43%)	37 (64%)
1–5 years	160 (16%)	7 (12%)	9 (16%)	7 (12%)
6–10 years	238 (24%)	14 (24%)	19 (34%)	9 (16%)
Above 10 years	40 (4%)	2 (3%)	4 (7%)	5 (9%)
Exclusive breastfeeding for 6 months	537 (54%)	33 (56%)	35 (63%)	30 (52%)
Vaccination				
Up‐to‐date vaccination^a^	378 (38%)	25 (42%)	28 (50%)	24 (41%)
Up‐to‐date PCV and pentavalent	512 (51%)	34 (58%)	39 (70%)	33 (57%)
History of symptoms as reported by mother				
Diarrhea	109 (11%)	3 (5%)	2 (4%)	5 (9%)
Fever	635 (64%)	37 (63%)	45 (80%)	51 (88%)
Cough	992 (99%)	59 (100%)	56 (100%)	58 (100%)
Fast/difficult breathing	674 (67%)	47 (80%)	36 (64%)	36 (62%)
Chest indrawing	20 (2%)	1 (2%)	1 (2%)	2 (3%)
URTI	661 (66%)	43 (73%)	19 (34%)	21 (36%)
Vomiting	35 (4%)	1 (2%)	0 (0%)	1 (2%)
Lethargy	1 (0%)	0 (0%)	0 (0%)	0 (0%)
Clinical and physical examination				
Temperature ≥ 37.5°C	319 (32%)	23 (39%)	29 (52%)	39 (67%)
Wheeze	37 (4%)	2 (3%)	4 (7%)	2 (3%)
Stunting (HAZ < −2 SD)	475 (48%)	25 (42%)	21 (38%)	34 (59%)
Wasting (WHZ < −2SD)	191 (19%)	10 (17%)	11 (20%)	7 (12%)
Underweight (WAZ < −2SD)	440 (44%)	24 (41%)	22 (39%)	30 (52%)
Oxygen saturation (%)				
90–92	167 (17%)	17 (29%)	8 (14%)	7 (12%)
93–95	277 (28%)	18 (31%)	19 (34%)	17 (29%)
> 95	556 (56%)	24 (41%)	29 (52%)	34 (59%)
Respiratory rate in infant (breaths/minute)	(*n* = 444)	(*n* = 33)	(*n* = 21)	(*n* = 19)
50–59	369 (83%)	28 (47%)	15 (27%)	14 (24%)
≥ 60	75 (15%)	5 (8%)	6 (11%)	5 (9%)
Respiratory rate in child (breaths/minute)	(*n* = 556)	(*n* = 26)	(*n* = 35)	(*n* = 39)
40–49	434 (78%)	20 (34%)	28 (50%)	32 (55%)
≥ 50	122 (19%)	6 (10%)	7 (13%)	7 (12%)
Anemia				
Some palmar pallor	92 (9%)	6 (10%)	1 (2%)	11 (19%)
Severe palmar pallor	0 (0%)	0 (0%)	1 (2%)	0 (0%)
Air quality indicators				
Regularly open windows for ventilation	860 (86%)	57 (97%)	47 (84%)	48 (83%)
Number of people sleeping in same room				
≤ 3	408 (41%)	23 (39%)	17 (30%)	19 (33%)
> 3	592 (59%)	36 (61%)	39 (70%)	39 (67%)
Pets in household	247 (25%)	13 (22%)	13 (23%)	10 (17%)
Current smoker in household	296 (30%)	18 (31%)	12 (21%)	22 (38%)
Type of fuel used for cooking				
Solid fuel	124 (12%)	11 (19%)	4 (7%)	3 (5%)
Natural gas	874 (87%)	48 (81%)	52 (93%)	55 (95%)
Kerosene oil	2 (0%)	0 (0%)	0 (0%)	0 (0%)
Type of cooking stove				
Close stove/modern sealed burners	43 (4%)	1 (2%)	1 (2%)	57 (98%)
Open fire stove/three‐brick stove	957 (96%)	58 (98%)	55 (98%)	1 (2%)
Place of cooking				
Part of main house (same room for living/sleeping)	93 (9%)	5 (8%)	5 (9%)	3 (5%)
Part of main house (separate room)	768 (77%)	36 (61%)	41 (73%)	49 (84%)
Outside house	139 (14%)	18 (31%)	10 (18%)	6 (10%)
Presence of child near cooking area	315 (32%)	17 (29%)	15 (27%)	17 (29%)

*Note*: An overview of the total sample size (*n* = 1000) and the distribution of key characteristics across three respiratory viruses (RSV, hMPV and influenza virus) is provided. The data are stratified by sex, age categories, maternal education status, breastfeeding practices, vaccination status, reported symptoms, clinical and physical examination findings, and household‐related factors.

Abbreviation: PCV, pneumococcal conjugate vaccine.

^a^ Children ≥ 3.5 months received vaccines doses as per the standard immunization schedule in Pakistan (OPV‐DPT‐Hep B‐Hib‐PCV‐Measles).

**TABLE 2:** Annual distribution of respiratory viruses in nasopharyngeal samples.

Year	No. of samples	Any virus positive (%)	RSV (%)	hMPV (%)	Influenza virus (%)
2015	682	625 (91.6)	36 (5.8)	33 (5.3)	32 (5.1)
2016	318	297 (93.4)	23 (7.7)	23 (7.7)	26 (8.7)
Total	1000	922 (92.2)	59 (6.4)	56 (6.0)	58 (6.3)

A history of fast/difficulty breathing was more common in children with RSV carriage (80%) compared to hMPV (64%) and influenza virus (62%). A recorded body temperature of ≥ 37.5°C was more commonly observed in children with influenza virus carriage (67%) compared to RSV and hMPV carriage (39% and 52%, respectively). Wheeze was infrequent overall but detected relatively more in children with hMPV carriage (7%) compared to RSV and influenza (3%). More than half of the children with influenza virus carriage were stunted and underweight as compared to children with hMPV and RSV carriage.

A higher proportion of children with RSV carriage had oxygen saturation of 90%–92% (29%) as compared to children with hMPV and influenza virus carriage. Among infants 2–11 months of age and children 12–59 months of age, almost a tenth of children with all three carriages had a respiratory rate ≥ 60 breaths per minute and a respiratory rate ≥ 50 per minute. Anemia, as observed by palmar pallor, was commonly seen in children with Influenza virus carriage (19%) compared to RSV (10%) and hMPV (2%). A majority of the children with RSV (61%), hMPV (70%), and influenza virus carriage (67%) shared their room with more than three individuals for sleeping, indicating overcrowding. Almost one in five children with RSV (22%), hMPV (23%), and Influenza virus carriage (17%) had pet animals, while a third had a smoker in the house. The majority of children with RSV, hMPV, and influenza virus carriage used natural gas as a source of fuel and open fire or three‐brick stoves for cooking.

Table 3 describes the predictors of RSV, hMPV, and influenza viral carriage. RSV carriage in children was positively associated with a history of fast/difficulty in breathing (aOR 1.96, 95% CI 1.02–3.76) and oxygen saturation of 90%–92% (aOR 2.52, 95% CI 1.32–4.82). HMPV carriage was positively associated with complete vaccination status (aOR 2.22, 95% CI 1.23–4.00) and temperature ≥ 37.5°C (aOR 2.34, 95% CI 1.35–4.04) whereas influenza virus carriage was associated with body temperature ≥ 37.5°C (aOR 4.48, 95% CI 2.53–7.93).

**TABLE 3:** Predictors of viral carriage in children with fast‐breathing pneumonia < 5 years of age.

Variables	OR	95% CI	*p*	aOR	95% CI	*p*
RSV carriage, *n* = 59						
History of symptoms as reported by mother						
Fast/difficult breathing	1.96	(1.02–3.76)	0.042	1.96	(1.02–3.76)	0.042
No fast/difficult breathing	Ref			Ref		
Oxygen saturation						
90%–92%	2.51	(1.31–4.80)	0.005	2.52	(1.32–4.82)	0.005
93%–95%	1.54	(0.83–2.89)	0.178	1.52	(0.81–2.86)	0.191
> 95%	Ref			Ref		
hMPV carriage, *n* = 56						
Sex						
Female	1.82	(1.04–3.22)	0.038	1.74	(0.99–3.04)	0.05
Male	Ref			Ref		
Vaccination						
Complete PCV and pentavalent vaccine^a^	2.28	(1.27–4.1)	0.006	2.22	(1.23–4.00)	0.008
Incomplete PCV and pentavalent vaccine	Ref			Ref		
Temperature (°C)						
< 37.5	Ref			Ref		
≥ 37.5	2.42	(1.4–4.16)	0.001	2.34	(1.35–4.04)	0.002
Influenza virus carriage, *n* = 58						
Temperature (°C)						
< 37.5	Ref			Ref		
≥ 37.5	4.85	(2.57–8.54)	< 0.001	4.48	(2.53–7.93)	< 0.05

*Note*: Odds ratios (OR) and adjusted odds ratios (aOR) with 95% confidence intervals (CI) for clinical and sociodemographic variables associated with respiratory syncytial virus (RSV), human metapneumovirus (hMPV), and influenza virus carriage are presented.

^a^ Children ≥ 3.5 months received vaccine doses as per the standard immunization schedule in Pakistan (OPV‐DPT‐Hep B‐Hib‐PCV‐Measles).

## 4 Discussion

We report that nearly two‐thirds of the children < 5 years old with fast‐breathing pneumonia had viral nasopharyngeal carriage in a peri‐urban community in Pakistan. Our findings are consistent with previous studies from LMICs, which confirm a high viral carriage prevalence in the nasopharyngeal niche among children with acute community acquired pneumonia [18]. Previously, Ali et al. reported 77.8% of the nasopharyngeal samples to be positive for viral carriage in rural Pakistan, and the most prevalent viruses detected was enterovirus/rhinovirus, followed by parainfluenza virus type III, and RSV [19]. RSV and hMPV exhibited higher detection rates in nasopharyngeal samples from hospitalized children aged 6 weeks to 2 years in another urban Pakistani setting [12]. A study from India focusing on hospitalized children under 14 years reported a 45.7% positivity rate for respiratory viruses in nasopharyngeal swabs [20]. Another study from India detected 15.4% influenza virus, 5% RSV, and 3.4% hMPV carriage in acute respiratory infection cases [21]. In Bangladesh, 77% of the pneumonia episodes in a birth cohort of children less than 2 years was associated with a respiratory virus [22]. Another study showed the predominance of RSV (13%), whereas hMPV and influenza virus were prevalent in 6% and 4% of the samples, respectively [23]. However, these studies were mostly carried out in children with severe pneumonia whereas fast‐breathing pneumonia is typically a milder and self‐limiting form of illness [11].

We observed a higher prevalence of viral carriage in other LMIC settings as compared to our findings. In a study from Iran, hMPV and RSV were detected in 15.7% and 30.0% of the samples, respectively, in children less than 5 years old. Another study from Iran reported 42.8% influenza carriage, 35.7% hMPV carriage, and 21.4% RSV carriage in pneumonia cases [24]. Among children from Morocco, 8.9% and 18.2% of the WHO‐defined severe pneumonia cases were infected with hMPV and RSV, respectively [25]. In Bulgaria, a total of 38% samples was influenza virus positive. RSV was detected in 15%, while hMPV was detected in 12% of the respiratory specimens [26]. In Greece, influenza viruses were detected in 28.3%, RSV in 10.4% samples, and hMPV in 6.5% of the children with acute respiratory infection [27]. In Thailand, influenza virus comprised 17.3% of the samples of which the majority were influenza A/H3N2. RSV and hMPV were identified in 11.4% and 3.6% of the samples, respectively [28]. In South Africa, 57.3% of the children with lower respiratory tract infection carried RSV, and influenza virus was present in 1.3% of these samples [29].

In our study, RSV carriage was positively associated with a history of fast/difficulty breathing and low oxygen saturation. This is consistent with previous studies which identify hypoxemia (90%–92% oxygen saturation) and difficult breathing with RSV carriage [30]. RSV has a higher propensity to involve lower respiratory airways than other infections [31]. A prospective RSV surveillance study in children of different age groups hospitalized with an acute respiratory infection (ARI) in a tertiary care hospital of Karachi, Pakistan, reported that children with RSV‐associated acute respiratory infection were almost four times more likely to be intubated as compared to non‐RSV infection [32]. Previously, Kazi et al. identified that deceased infants who exhibited respiratory symptoms during their illness were more likely to test positive for RSV compared to those who had no respiratory symptoms. Pulse oximetry (%Sat) was reliably associated with the need for oxygen therapy [33]. This reflects its practical utility for assessing oxygenation in children, given its cost‐effectiveness and ease of use in community settings. Consistent with our findings, Kazi et al. reported that RSV carriage and deaths mostly occurred in younger age groups, particularly within the 0–3 months. Young infants are at a higher risk of severe outcomes including lower respiratory tract disease and death and can be a target for potential RSV vaccine. In addition, maternal RSV vaccination could offer protection through maternally acquired RSV antibodies in the first 3–6 months of life [33].

In our study, hMPV carriage in children was positively associated with fully vaccinated status, and fever, while influenza viral carriage was only associated with fever [34]. Fever in hMPV and influenza viral infection suggests the presence of an infection with an inflammatory response elicited by the body [35]. HMPV often causes pneumonia and other viral or bacterial coinfections in children. In a previous study from Pakistan, hMPV was detected in 7% of the children with community acquired pneumonia, a majority of these children reported cough (86%), fever (73%), nasal congestion (69%) and shortness of breath (68%). Although previous literature has shown a significantly high frequency of pneumococcal nasopharyngeal carriage in hMPV‐infected patients [36], we found hMPV carriage to be positively associated with a complete PCV and Pentavalent vaccination status. This could be due to the ecological niche being taken over by viral pathogens in the post‐bacterial vaccine era. Consistent with these findings, previous studies have reported 65% of the nonsevere/fast‐breathing community acquired pneumonia to be viral in etiology with a bacterial‐viral coinfection in about 30% of children after the introduction of the Pneumococcal Conjugate Vaccines [5]. Nasir et al. identified a history of fever and cough to have highest predictive value which could correctly diagnose influenza in over 60%–70% patients on the basis of clinical symptoms alone [37]. However, fever is a nonspecific sign, and all three of these viruses often present with similar cold‐like symptoms. Our study attempts at pinpointing minor differences in their clinical characteristics. Children with hMPV carriage had an increased history of fever and cough. In contrast, RSV‐infected patients exhibited higher rates of fast and difficult breathing and low oxygen saturation whereas children carrying influenza virus had a higher frequency of diarrhea, fever, cough, and nutritional deficiencies such as anemia, stunting, and wasting.

The exact burden of respiratory viruses such as RSV, hMPV, and influenza virus in Pakistan remains unknown; most literature is hospital‐based, which underestimates their true prevalence in the community. Ibrahim‐Hyderi is a peri‐urban low‐income community with a high Infant mortality rate (IMR) of 60.1, and an under‐five mortality rate (U5MR) of 69.5 per 1000 live births, a majority of which is attributable to vaccine preventable respiratory infections [38]. Seventy‐six percent of the population refuses to seek hospital‐based care due to financial constraints, language barriers, religious, and cultural beliefs, so community‐acquired RSV, hMPV and influenza virus infections may never be tested or treated in hospitals. A previous study showed that healthcare seeking was higher in this community when the mother recognized the severity of the illness, presence of respiratory distress, and temperature < 35.5 °C [39]. Thus, a symptom‐based case definition can help community workers refer sick children for testing appropriately.

We investigated the predictors of RSV, hMPV, and influenza virus among children with fast‐breathing pneumonia in Pakistan. While these viruses share a similar clinical presentation and collectively constitute the most prevalent causes of severe pneumonia in LMICs, as highlighted by the PERCH study, their differences exist, underscoring the importance of discerning predictors for each virus [35]. The widespread unavailability and affordability constraints of molecular viral testing in many clinical settings pose challenges in isolating the specific virus etiology. In Pakistan, influenza vaccines are available in the private market although the coverage remains suboptimal, whereas RSV and hMPV lack specific vaccines. Our findings offer preliminary insights into the typical clinical presentation of RSV, hMPV, and influenza virus infections in children. However, the sensitivity of clinical predictors for viral respiratory infections depends on a multitude of factors including prevalence of disease, age, underlying illnesses, duration of symptoms prior to consultation, and the vaccination status in the population being evaluated. These identified signs and symptoms could be used to inform a targeted disease‐based algorithm such as the IMCI for use by community health workers, improving their ability to diagnose specific viral infections.

Our study had several strengths; we performed a prospective data collection with analysis using a qualitative multiplex molecular diagnostic assay, Luminex xTAG RVP, for viral detection in children with mild pneumonia. The data were collected prior to the emergence of the COVID‐19 pandemic. As the landscape of respiratory infections has evolved significantly in the post‐COVID period, we believe interpreting these findings in the current context requires caution. The study was performed in a peri‐urban community and may have limited generalizability to other settings. We could only enroll a subset of the participants due to limited resources available. We could not explore bacterial coinfections and viral load, which could be representative of an early progression and a more severe disease presentation. This was beyond the scope of the study and requires further examination. Viral nasopharyngeal carriage does not always equate to a viral etiology and this situation is further complicated by limited diagnostics and difficulty in obtaining appropriate lower respiratory tract specimen. The lack of a control group or nasopharyngeal swabs from healthy children in our study was also a limitation.

## 5 Conclusion

We observed a high nasopharyngeal viral carriage among children with WHO‐defined fast‐breathing pneumonia Rhinovirus was identified as the most prevalent virus, followed by RSV, hMPV, and influenza virus. We identified fever, difficulty breathing, hypoxia, and a complete vaccination status as important clinical predictors for viral CAP. The findings also highlight the use of a symptom‐based approach for efficient testing and referral especially in resource‐constrained settings and potential vaccine development to mitigate the impact of specific viral pathogens in this community.

## Author Contributions

**Muhammad Imran Nisar:** Conceptualization; Data curation; Formal analysis; Funding acquisition; Investigation; Methodology; Project administration; Resources; Software; Supervision; Validation; Visualization; Writing – original draft; Writing – review and editing. **Salima Kerai:** Data curation; Formal analysis; Investigation; Software; Validation; Visualization; Writing – original draft; Writing – review and editing. **Shahira Shahid:** Formal analysis; Methodology; Software; Validation; Visualization; Writing – original draft; Writing – review and editing. **Muhammad Farrukh Qazi:** Formal analysis; Software; Visualization; Writing – review and editing. **Sarah Rehman:** Formal analysis; Investigation; Methodology; Validation; Visualization; Writing – original draft; Writing – review and editing. **Fatima Aziz:** Formal analysis; Investigation; Methodology; Resources; Validation; Visualization; Writing – original draft; Writing – review and editing. **Fyezah Jehan:** Conceptualization; Data curation; Formal analysis; Funding acquisition; Investigation; Methodology; Project administration; Resources; Software; Supervision; Validation; Visualization; Writing – original draft; Writing – review and editing.

## Conflicts of Interest

The authors declare no conflicts of interest.

### Peer Review

The peer review history for this article is available at https://www.webofscience.com/api/gateway/wos/peer‐review/10.1111/irv.13285.

## Data Availability

The dataset for this manuscript will be available from the corresponding author upon request.
